# The Spectrum of Gallbladder Histopathology at a Tertiary Hospital in a Developing Country: A Retrospective Study

**DOI:** 10.7759/cureus.9627

**Published:** 2020-08-09

**Authors:** Talal Almas, Muhammad Faisal Murad, Muhammad Kashif Khan, Muneeb Ullah, Faisal Nadeem, Maryam Ehtesham, Syed Muhammad Jawad Zaidi

**Affiliations:** 1 Internal Medicine, Royal College of Surgeons in Ireland, Dublin, IRL; 2 General Surgery, Maroof International Hospital, Islamabad, PAK; 3 Surgical Oncology, Federal Government Poly Clinic (Post Graduate Medical Institute), Islamabad, PAK; 4 Surgical Oncology, Maroof International Hospital, Islamabad, PAK; 5 Laparoscopic Surgery, Maroof International Hospital, Islamabad, PAK; 6 Internal Medicine, Rawalpindi Medical University, Rawalpindi, PAK

**Keywords:** gallbladder, chronic cholelithiasis, xanthogranulomatous, histopathology

## Abstract

Background

Affections of the gallbladder remain exceedingly ubiquitous and often warrant surgical intervention. The histopathological patterns represent a spectrum, ranging from cholecystitis to gallbladder carcinoma. The present study aims to delineate the occurrence of various gallbladder histopathologies in a tertiary care hospital in Pakistan.

Methods

A retrospective study was conducted at Maroof International Hospital, Islamabad, Pakistan. Histopathological records of 442 gallbladder specimens obtained from cholecystectomy were analysed. The prevalence of various histopathological outcomes was assessed. The data were eventually analysed using the SPSS 23.0 software (Armonk, NY: IBM Corp.). Thereafter, the distribution of various gallbladder histopathologies was tabulated across gender.

Results

Of the 442 patients included, 330 were females and 112 were males, with the mean age hovering at 45.77±14.65 years. The most common histopathological findings were chronic cholecystitis and cholesterolosis, observed in 78.6% and 32.8% of the patients, respectively. While only one case of gallbladder adenocarcinoma was observed, multiple specimens divulged premalignant lesions including reactive atypia and intestinal metaplasia.

Conclusions

Diseases of the gallbladder often mandate prompt surgical intervention. Of these, chronic cholecystitis, which is an established risk factor for gallbladder carcinoma, is exceedingly common. The employment of histopathological techniques remains imperative in the detection of premalignant and malignant lesions that might otherwise evade macroscopic detection and thus progress to adenocarcinoma.

## Introduction

The spectrum of ailments that afflict the gallbladder (GB) can be inflammatory, congenital, or neoplastic in nature [1,2]. The signs and symptoms elicited by these pathologies evoke the need for surgical intervention through the means of either open or laparoscopic cholecystectomy. Inflammatory conditions of the GB are noted to be more common than other GB pathologies, and encompass a spectrum of ailments, including acute, chronic, follicular, or xanthogranulomatous cholecystitis (XGC) [3,4]. While carcinoma of the GB remains a relatively rare aetiology, ubiquitous conditions, such as chronic cholecystitis secondary to gallstones, can irritate the GB mucosa, thereby heralding the onset of metaplastic and dysplastic transformations. These transformations can, in turn, predispose the patients to the future development of gallbladder carcinoma (GBC) [4,5]. Timely cholecystectomy to treat such longstanding inflammatory conditions therefore remains focal. Additionally, due to the possibility of an underlying, clinically silent malignancy or a premalignant lesion, meticulous histopathological evaluation of the resected GB specimen remains imperative [6]. The carcinoma of the GB is a rare but clinically silent disease that is most commonly associated with longstanding cholelithiasis [7]. In patients with longstanding, chronic cholecystitis, a suspicion of malignancy often warrants thorough histopathological workup of the resected specimen [6,7]. Pertinently, dilatory detection of GB cancer can lead to adverse outcomes for the patient [8]. Even in instances where it is detected early, GBC boasts a forbidding mortality rate [5,8,9]. To prevent these grave complications, histopathological workup of resected specimens is warranted in order to thwart the pathogenesis underlying GBC. 

In Pakistan, the practice of conducting a histopathological analysis of GB specimens at peripheral hospital setups lags due to insufficient resources and an exorbitant patient load [10,11]. In a myriad of medical institutions in Pakistan, only the specimens remarkable for macroscopic abnormalities are sent for histopathological evaluation [10,12]. Interestingly, this practice stands in stark contrast to the globally touted practices that vouch for the due evaluation of most GB specimens in order to discern the presence of an underlying malignant or premalignant lesion [12]. Furthermore, histopathological workup facilitates the prompt detection of premalignant lesions that might appear macroscopically unremarkable. The overarching aim of this study is to elucidate the spectrum of histopathological ailments that most commonly affect the GB and thereby warrant surgical intervention. Additionally, the study also aims to evaluate the distribution of the various GB morphological variants with respect to the gender of the patients.

## Materials and methods

A retrospective study was conducted in the department of surgery, Maroof International Hospital, Islamabad, Pakistan. GB specimens were obtained from cholecystectomies performed (both laparoscopic and open) during the study period of January, 2017 to December, 2019. The results obtained from the histopathological workup were then analysed. Histopathology reports that were either unavailable or yielded insufficient information for any reason were excluded from the study sample. Keeping in mind the exclusion criteria, a total of 442 histopathology reports were included and subsequently analysed with regards to the distribution of GB disease across gender and different age groups. The data were ultimately analysed using the SPSS 23.0 software (Armonk, NY: IBM Corp.). 

## Results

In the present study involving 442 cases, 330 and 112 samples were obtained from female and male patients, respectively. The male-to-female ratio was approximately 1 to 3. The mean age of the patients included in the study was 45.77±14.65 years. Most of the patients who underwent cholecystectomy for gallstone disease were noted to present between the fourth and sixth decades of their lives. The breakdown of the participants with pertinence to gender is tabulated in Table 1.

**Table 1:** A depiction of the breakdown of the study participants with respect to gender.

Gender	Frequency	Percentage
Male	112	25.3%
Female	330	74.7%

Furthermore, a breakdown of the study population with respect to age is delineated in Table 2. 

**Table 2:** A delineation of the breakdown of study participants in accordance with age.

Age groups (years)	Frequency	Percentage
5-25	30	6.8%
26-45	196	44.3%
46-65	177	40%
66-75	31	7%
>75	8	1.8%

A total of 348 patients, accounting for 78.7% of the total patients, demonstrated chronic cholecystitis, which appears to be the most common histopathological feature in our study. Additionally, cholesterolosis and acute-on-chronic cholecystitis were also common in our study population. Imperatively, there was only one case of adenocarcinoma of the GB. The frequencies of the various histopathological features and their distribution with respect to gender are delineated in Table 3. 

**Table 3:** The diverse spectrum of the histopathological outcomes observed in the study population with respect to gender.

Histopathological features	Frequency		
	Males (n=112)	Females (n=330)	Total
Acute cholecystitis	3	8	11 (2.5%)
Acute-on-chronic cholecystitis	28	44	72 (16.3%)
Adenocarcinoma	0	1	1 (0.2%)
Cholesterolosis	13	132	145 (32.8%)
Chronic cholecystitis	80	268	348 (78.7%)
Empyema gallbladder	1	4	5 (1.1%)
Follicular cholecystitis	0	2	2 (0.5%)
Intestinal metaplasia	0	1	1 (0.2%)
Lymphocytic cholecystitis	0	1	1 (0.2%)
Polyp	1	0	1 (0.2%)
Reactive atypia	1	1	2 (0.5%)
Xanthogranulomatous cholecystitis	1	4	5 (1.1%)

## Discussion

Cholecystectomy is a widely performed surgical procedure and is implicated in the management of a multitude of pathologies, including cholelithiasis, cholecystitis, GB polyps, and GBC. Amongst these aforesaid pathologies, cholelithiasis constitutes the most prevalent biliary tract pathology globally with a prevalence rate hovering at 10%-15% [13]. While benign themselves, gallstones have been intricately linked with an increased risk of hepatobiliary and GBC [14]. Although the prevalence of GBC in the general population is meagre, GBC constitutes 80% of all biliary tract cancers [15]. Due to its late presentation in the disease course, GBC purports a particularly poor prognosis and dismal five-year survival rates [16]. 

In the present study, a female predominance amongst the patient population was observed. In concert with this finding, a study conducted in India, a neighbouring nation, concluded a male-to-female ratio of 1:2.4 [17,18]. Interestingly, the female gender is noted to be a risk factor that predisposes to the development of gallstones. Almost all of the patients in our study presented with cholelithiasis, which can eventually herald the onset of various pathologies, such as acute cholecystitis, chronic cholecystitis, follicular cholecystitis, and cholesterolosis. The prevalence of chronic cholecystitis, the most common pathology within our patient population, was noted to be 78.7%. In accordance with this finding, a study concluded the prevalence of chronic cholecystitis to be 79.8%, which bears remarkable semblance to our finding [19]. Chronic cholecystitis is characterised by marked thickening of the GB wall with associated calcifications, eventually culminating in a pathology termed the porcelain GB. The presence of porcelain GB is an established risk factor for the development of a GBC [7].

Cholesterolosis results from mucosal villous hypertrophy and a resultant accumulation of cholesterol esters and triglycerides in a diffuse or polypoid form in the macrophages present within the wall of the GB. In our study, cholesterolosis was observed in 32.8% of the cases. A study conducted by Mondal et al. concluded the presence of the pathology in merely 2.9% of the patient population, while Sangwan et al. reported a prevalence of 9.43% [5,19]. The prevalence of the pathology in our study was therefore noted to be significantly higher. 

XGC, a chronic, focal, or diffuse fibroinflammatory process resulting from an intramural accumulation of foamy histiocytes, was observed in 1.1% of the cases. XGC exhibits preoperative and intraoperative findings similar to those manifested by GBC [20]. XGC presents with pericholecystic infiltration, hepatic involvement, and lymphadenopathy; its diagnosis can therefore remain elusive. A delayed diagnosis of XGC mandates the need for an extraneous radical surgery rather than the standard cholecystectomy [21]. Moreover, its association with GBC has also been well established [19,21]. 

Empyema of the GB is a ramification of acute cholecystitis and is noted to occur in 5%-15% of cases [22]. Clinically, the pathology presents as a pus-filled, distended GB with histopathological findings significant for marked edema, inflammation, and fibrinous exudate surrounding the wall of the GB [22]. In this study, empyema of the GB was observed in 1.1% of the cases, which is similar to a prevalence rate of 0.71% as purported by another study [6].

Intestinal metaplasia and reactive atypia were observed in 0.2% and 0.5% of the cases, respectively. While their prevalence in the current study was indubitably low, their malignant potential merits a consideration. Intestinal metaplasia and reactive atypia are both premalignant conditions that can eventually lead to the development of carcinoma. Imperatively, most of the patients who presented with these premalignant afflictions belonged to the older age groups. This indicates that an increased age at presentation increases the risk of a malignant transformation [5,15]. If not detected early, these lesions can orchestrate the pathogenesis that underlies the development of GBC. It is therefore necessary to evaluate the histopathology of each GB specimen, irrespective of its macroscopic appearance intraoperatively. Doing so can aid the early detection of carcinoma in high-risk patients, thus curbing the risk of progression to advanced disease. 

GB adenocarcinoma was observed in merely 0.2% of the cases. Various studies have reported prevalence rates ranging from 0.5% to 1.05% of the total cases [7,23]. Despite the advent of modern diagnostic techniques, GBC is still diagnosed at a late stage and is thus associated with a poor prognosis [23]. Furthermore, since GBC often remains clinically silent in its initial stages, it evades prompt detection. Nevertheless, histopathological examination, ameliorated imaging techniques, and specific diagnostic markers can abet in yielding a timely diagnosis, thereby improving disease outcomes. 

## Conclusions

GB disease remains a major indication for cholecystectomy. Postoperative histopathological evaluation of the excised GB specimens divulges a vast spectrum of underlying pathologies. Of these, chronic cholecystitis, cholesterolosis, and acute-on-chronic cholecystitis remain the most prevalent. Furthermore, a macroscopic absence of remarkable features does not preclude the presence of an underlying premalignant or malignant lesions. There is thus an overarching need for routine histopathological examination of the resected GB specimens in order to exclude premalignant ailments such as intestinal metaplasia and reactive atypia. Left undetected, these lesions can progress to GB adenocarcinoma, which is noted to have a particularly forbidding prognosis.
